# Differential Regulation of the Extracellular Cysteine/Cystine Redox State (E_h_CySS) by Lung Fibroblasts from Young and Old Mice

**DOI:** 10.1155/2016/1561305

**Published:** 2016-08-25

**Authors:** Walter H. Watson, Tom J. Burke, Igor N. Zelko, Edilson Torres-González, Jeffrey D. Ritzenthaler, Jesse Roman

**Affiliations:** ^1^Department of Medicine, Division of Gastroenterology, Hepatology and Nutrition, University of Louisville School of Medicine, Louisville, KY, USA; ^2^Department of Pharmacology and Toxicology, University of Louisville School of Medicine, Louisville, KY, USA; ^3^Department of Biochemistry and Molecular Genetics, University of Louisville School of Medicine, Louisville, KY, USA; ^4^Department of Medicine, Division of Pulmonary, Critical Care, and Sleep Medicine, University of Louisville School of Medicine, Louisville, KY, USA; ^5^Robley Rex Veterans Affairs Medical Center, Louisville, KY, USA

## Abstract

Aging is associated with progressive oxidation of plasma cysteine (Cys)/cystine (CySS) redox state, expressed as E_h_CySS. Cultured cells condition their media to reproduce physiological E_h_CySS, but it is unknown whether aged cells produce a more oxidized extracellular environment reflective of that seen in vivo. In the current study, we isolated primary lung fibroblasts from young and old female mice and measured the media E_h_CySS before and after challenge with Cys or CySS. We also measured expression of genes related to redox regulation and fibroblast function. These studies revealed that old fibroblasts produced a more oxidizing extracellular E_h_CySS than young fibroblasts and that old fibroblasts had a decreased capacity to recover from an oxidative challenge due to a slower rate of reduction of CySS to Cys. These defects were associated with 10-fold lower expression of the Slc7a11 subunit of the xCT cystine-glutamate transporter. Extracellular superoxide dismutase (Sod3) was the only antioxidant or thiol-disulfide regulating enzyme among 36 examined that was downregulated in old fibroblasts by more than 2-fold, but there were numerous changes in extracellular matrix components. Thus, aging fibroblasts not only contribute to remodeling of the extracellular matrix but also have a profound effect on the extracellular redox environment.

## 1. Introduction

Aging is associated with increased incidence of respiratory disorders, and elderly patients represent a disproportionate number of afflicted individuals with pneumonia, acute lung injury, and lung fibrosis, among other lung disorders [1–5]. This association has also been noted in experimental models of lung disease. For example, senescent rodent lungs are more susceptible to lung injury in the setting of mechanical ventilation, ozone exposure, and pulmonary infection [6–10]. Furthermore, intratracheal instillation of lipopolysaccharide results in the exaggerated expression of proinflammatory cytokines in senescent animals when compared to young controls [9, 11]. Senescent lungs are also more susceptible to bleomycin-induced lung injury [12, 13]. Together, these studies point to the enhanced susceptibility of the senescent lung to injury, but little is known about the factors responsible for this susceptibility.

Several mechanisms have been proposed to explain the abovementioned observations including increased oxidative stress and free radical damage, a decline in immune responses, and alterations in stem cell/progenitor cell differentiation potential [14–16]. Mitochondrial dysfunction has also been implicated in aging since the coordination between nuclear and mitochondrial communication during aging appears to be affected [17]. There is also much literature showing alterations in lung structure and function in the aging lung [18–21]. Consistent with this, we previously reported that aging murine lungs harvested from old mice are characterized by increased expression of fibronectin and collagen matrix mRNAs and by induction of the profibrotic factor, transforming growth factor *β* [22]. Thus, active matrix remodeling may account for the subtle changes observed in lung structure as well as the decline in lung function observed in the elderly [23] and might render the host susceptible to disrepair after lung injury [22].

Another abnormality associated with aging is a shift in the redox states of plasma thiol-disulfide redox couples [24, 25]. Cysteine (Cys) and its disulfide cystine (CySS) constitute the major small molecular weight thiol-disulfide redox couple in extracellular compartments. The redox state of the Cys/CySS couple, expressed as E_h_CySS calculated from the Nernst equation, is about −80 mV in the plasma of healthy young adults [26] but is more oxidized in older individuals [24]. The mechanisms that regulate E_h_CySS and the factors that lead to its oxidation with aging are largely unknown.

Accumulating evidence suggests that cells actively participate in the control of their extracellular redox environment. When mammalian cells are grown in culture, some of the CySS that is provided by the medium is converted to Cys until the optimal redox state of −80 mV is reached [27–29]. If one replaces the CySS in the medium with Cys, the cells will again adjust the concentrations of extracellular Cys and CySS to achieve a redox state of −80 mV. These observations suggest that there is an optimal set point for the extracellular redox state and that cells have the capacity to restore this equilibrium when challenged with either reducing or oxidizing conditions.

Fibroblasts play a key role in maintaining the extracellular matrix, but it is unknown if they contribute to the maintenance of the extracellular redox environment as well. We previously reported that primary lung fibroblasts cultured in media with oxidized E_h_CySS showed increased expression of fibronectin and TGF*β*1, among other changes, indicating that oxidation of the E_h_CySS alters matrix gene expression in lung fibroblasts [30]. The purpose of the present study was to determine whether fibroblasts from old and young mice have different extracellular E_h_CySS set points and whether these differences translate into altered expression of extracellular matrix-related genes.

## 2. Materials and Methods

### 2.1. Reagents

All reagents were purchased from Sigma Chemicals (St. Louis, MO) or Fisher Scientific (Pittsburgh, PA) unless otherwise specified.

### 2.2. Animals and Primary Lung Fibroblasts

Mice were housed in a pathogen-free barrier facility accredited by the Association for Assessment and Accreditation of Laboratory Animal Care, and procedures were approved by the University of Louisville's Institutional Animal Care and Use Committee. The animals used in this study were young (2 months old) or old (24 months old) female C57BL/6 mice purchased from Charles River Laboratories (Wilmington, MA). Primary lung fibroblasts were harvested from these animals and cultured in DMEM supplemented with 10% FBS and 1% antibiotic-antimycotic solution (Cellgro, Manassas, VA) as previously described [31, 32]. All experiments described here used cells between passages numbers 3 and 8.

### 2.3. Preparation of Media with Different E_h_ Cys/CySS Redox States

Media with redox states of 0, −80, and −150 mV were prepared in cyst(e)ine-free DMEM by adding different concentrations of Cys and CySS while keeping the total concentration ([Cys] + 2 *∗* [CySS]) constant at 200 *μ*M. For 0 mV media, [Cys] = 0.5 *μ*M and [CySS] = 99.75 *μ*M. For −80 mV, [Cys] = 14 *μ*M and [CySS] = 93 *μ*M, and for −150 mV, [Cys] = 130 *μ*M and [CySS] = 35 *μ*M. Redox media were added to cells within 10 minutes of preparation.

### 2.4. Culturing Cells in Redox Media

Primary lung fibroblasts from young mice and old mice were plated in 6-well plates at a density of 8 × 10^5^ cells/well in DMEM supplemented with 10% FBS and 1% antibiotic-antimycotic solution. After 24 hours, the media on all plates were replaced with serum-free media containing 4 mM L-glutamine, 1% antibiotic-antimycotic solution, and Cys and CySS at concentrations that produced one of 3 different E_h_CySS: oxidizing (0 mV), typical (−80 mV), or reducing (−150 mV). Aliquots of each media were added to an empty 6-well plate (for 0 hours no-cell controls) or to plates containing young and old cells (for 0 hours time points) and immediately removed and processed for Cys/CySS and GSH/GSSG analysis. Later time points were collected after incubation of plates containing young, old, or no cells for the indicated times.

### 2.5. Sample Collection and Analysis of Cys, CySS, GSH, and GSSG

Conditioned media were centrifuged at 500 ×g to pellet any unattached cells. The cell-free media were immediately transferred to a centrifuge tube containing an equal volume of ice-cold 10% (w/v) perchloric acid, 0.2 M boric acid, and 20 *μ*M *γ*-glutamyl glutamate as an internal standard [33]. Extracts were centrifuged at 16,000 ×g for 2 minutes to remove precipitated protein. The protein-free extracts were derivatized with iodoacetic acid and dansyl chloride and analyzed by HPLC (Waters Corporation, Millford, MA) as previously described [34]. Concentrations of thiols and disulfides were determined by integration relative to the internal standard. Redox potentials (E_h_) of the GSH/GSSG and Cys/CySS pools, given in millivolts (mV), were calculated from concentrations of GSH, GSSG, and Cys and CySS in molar units with the following forms of the Nernst equation for pH 7.4: E_h_ GSH/GSSG = −264 + 30*∗*log⁡([GSSG]/[GSH]^2^); E_h_ Cys/CySS = −250 + 30*∗*log⁡([CySS]/[Cys]^2^)  [26].

### 2.6. Microarray Analysis

Biotinylated cRNA was prepared from total RNA using Affymetrix GeneChip Whole Transcript (WT) Plus Reagent kit. Fragmented cRNA was hybridized to Affymetrix MoGene-2_0-st-v1 arrays and processed on an Affymetrix FS-450 fluidics station and scanned on an Affymetrix GeneChip scanner. The resulting  .cel files were imported into Partek Genomics Suite 6.6 (6.15.0327) and transcripts were normalized and summarized using RMA default settings. Samples were compared using a one-way ANOVA model to assess the contribution of age. A step-up false discovery rate was applied as multiple test correction for the resulting *p* values.

### 2.7. Messenger RNA Expression Analysis

RNA was isolated from primary mouse lung fibroblasts utilizing the Quick-RNA MicroPrep kit from Zymo Research (Irvine, CA). cDNA was synthesized using the iScript cDNA Synthesis Kit (Bio-Rad, Hercules, CA). Real Time RT-PCR was performed with the following mouse primer sets: 18S forward (5′-GGA CCA GAG CGA AAG CA), reverse (5′-ACC CAC GGA ATC GAG AAA); Gapdh forward (5′-CAG CCT CGT CCC GTA GAC AA), reverse (5′-TTT GAT GTT AGT GGG GTC TC); EDA fibronectin forward (5′-TGT ATG CTG TCA CTG GCC), reverse (5′-GTT GAT TTC TTT CAT TGG TCC); Sod3 forward (5′-AGG TGG ATG CTG CCG AGA T), reverse (5′-TCC AGA CTG AAA TAG GCC TCA AG). Amplifications were performed in the Cepheid Smart Cycler (Sunnyvale, CA) using iTaq Universal SYBR Green Supermix (Bio-Rad, Hercules, CA). The following cycle program was used: hold 95°C for 120 s; 40 cycles at 95°C for 15 s, and 64°C for 30 s. To analyze mRNA levels of Slc7a11, GPx7, and Rn18S, real time PCR was performed with TaqMan probes (TaqMan® Gene Expression Assay Mm00442530_m1, Mm00481133_m1, and Mm03928990_g1; Applied Biosystems), according to the manufacturer's protocol (TaqMan Universal Master Mix II; Applied Biosystems). The PCR was performed utilizing the Step One Plus Real Time PCR System (Applied Biosystems) with the parameters: 1 cycle of 50°C 2:00 min, 95°C 10:00 min, followed by 40 cycles of 95°C 00:15 sec, 60°C 1:00 min. Results were analyzed using Step One Software version 2.3 (Applied Biosystems). For all PCR reactions, the amplification curves were analyzed by the mathematical equation of the second derivative. The amount of target mRNA expression was normalized to the 18S housekeeping gene, and relative quantification was performed using the 2^−ΔΔCT^ method [35].

### 2.8. Statistical Analysis

All data are expressed as mean ± standard deviation. Unpaired two-tailed *t*-tests and one-way analysis of variance tests were used for single and multiple comparisons, respectively (*p* values < 0.05 were considered significant). Posttest analysis was performed using Dunnett's multiple comparison test to compare between groups. GraphPad Prism and GraphPad In-Stat version 4 software (GraphPad Software, Inc., La Jolla, CA) were used to calculate the statistics.

## 3. Results

### 3.1. Extracellular E_h_CySS Is Regulated Differently by Lung Fibroblasts from Young and Old Mice

**Figure 1:**
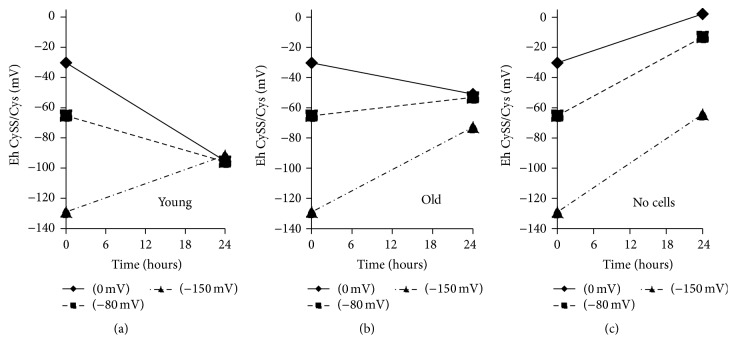
E_h_CySS in media conditioned by fibroblasts isolated from the lungs of young and old mice. Primary lung fibroblasts from young mice and old mice were cultured in one of 3 different redox media: oxidizing (0 mV), typical (−80 mV), or reducing (−150 mV). Media was removed immediately (0 hours) or after 24 hours and processed for determination of Cys and CySS concentrations and E_h_CySS as described in Section 2. (a) Change in E_h_CySS of 3 different redox media following 24-hour incubation with cells from young mice. (b) Change in E_h_CySS of 3 different redox media following 24-hour incubation with cells from old mice. (c) Change in E_h_CySS of 3 different redox media following 24-hour incubation in the absence of cells.

Fibroblasts isolated from the lungs of a young mouse conditioned the culture medium to achieve an extracellular E_h_CySS of about −95 mV within 24 hours, independent of the starting redox potential of the medium (Figure 1(a)). In contrast, cells from old mice challenged with oxidizing media (0 mV) or with physiological redox media (−80 mV) stabilized at a redox potential that was about 40 mV more oxidizing than that achieved by young cells (Figure 1(b)). When old cells were challenged with a reducing environment (−150 mV), they did not quite return to the same level as when cultured in either oxidizing (0 mV) or typical (−80 mV) media but were still more oxidizing than was seen in the young cell cultures.

**Figure 2:**
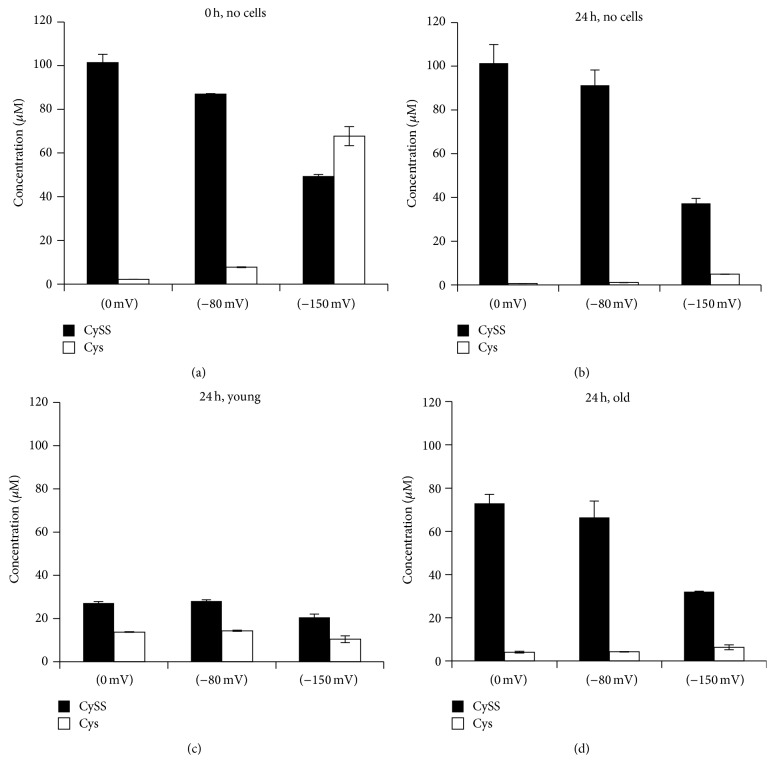
Cys and CySS concentrations in media conditioned by fibroblasts isolated from the lungs of young and old mice. (a) Cys and CySS concentrations in redox media at time 0 hours and (b) after 24 hours of incubation at 37°C in the absence of cells. (c) Cys and CySS concentrations in the conditioned media of fibroblasts from young and (d) old mice after 24 hours of incubation at 37°C.

In the absence of cells, all 3 redox media became more oxidized over the 24-hour incubation period (Figure 1(c)). CySS concentrations were quite stable in all 3 media over 24 hours, but Cys concentrations dropped dramatically. Thus, oxidation of the media redox potential in the absence of cells reflected a decrease in Cys rather than an increase in CySS (Figures 2(a) and 2(b)). The pattern of changes in CySS and Cys was different in the presence of cells. In the presence of young cells, CySS concentrations in the conditioned media were lower than in the presence of old cells (Figures 2(c) and 2(d)). CySS concentrations were decreased by 60 to 75% over 24 hours in the young cell culture media, whereas there was only a 30% decrease in CySS in the old cell media.

Some of the CySS in both young and old cultures appeared to be metabolized to Cys. Cys concentrations in conditioned media from both young and old cell cultures, and in all 3 redox media, were higher than the Cys concentrations seen in the corresponding redox media incubated in the absence of cells (compare Figures 2(c) and 2(d) to 2(a) and 2(b)). Both young and old cells produced Cys at rates that exceeded the spontaneous degradation observed in the absence of cells. However, old cells reached a lower steady state level of Cys than young cells.

### 3.2. Rate of CySS Consumption Is Slower in Old Cells Than in Young Cells

**Figure 3:**
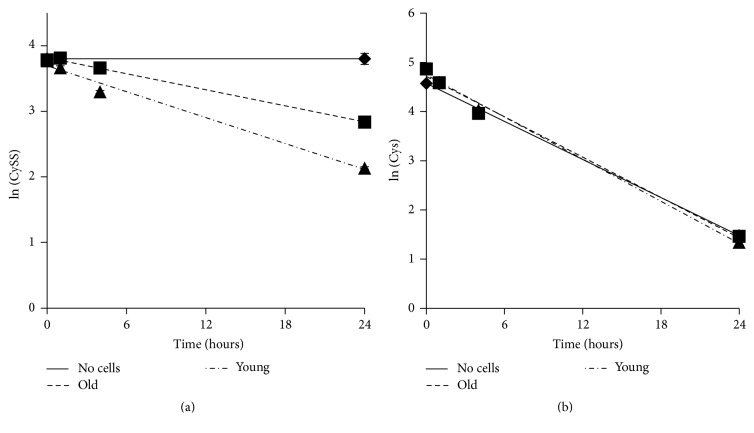
Extracellular CySS is consumed more rapidly by fibroblasts from young mice than by fibroblasts from old mice. DMEM containing 45 *μ*M CySS (a) or 100 *μ*M Cys (b) was added to lung fibroblasts from young and old mice. Media were removed at different time points for analysis of Cys and CySS concentrations by HPLC. Concentrations were transformed as the natural logarithm. The line through the data represents the best fit via linear regression.

The data in Figure 2 suggested that young cells removed CySS from the media more rapidly than old cells. To investigate this further, we measured CySS concentrations in media to which CySS was added as a function of incubation time. Although CySS did not change in the absence of cells, there was an exponential decline in extracellular CySS in both the young and old cell cultures (Figure 3(a)). The data were plotted as the natural log of the CySS concentration, and the slope of the line was used to calculate the half-life of CySS in each culture. Accordingly, media on young cells exhibited a CySS half-life of 10.5 hours, whereas media on old cells had a CySS half-life of 17.0 hours. In contrast, the half-life of Cys in media was 5 hours, and the presence of either young or old cells had no effect on the stability of Cys (Figure 3(b)).

### 3.3. The Rate of Equilibration of the Extracellular Redox Environment Is Slower in Old Cells Than in Young Cells

**Figure 4:**
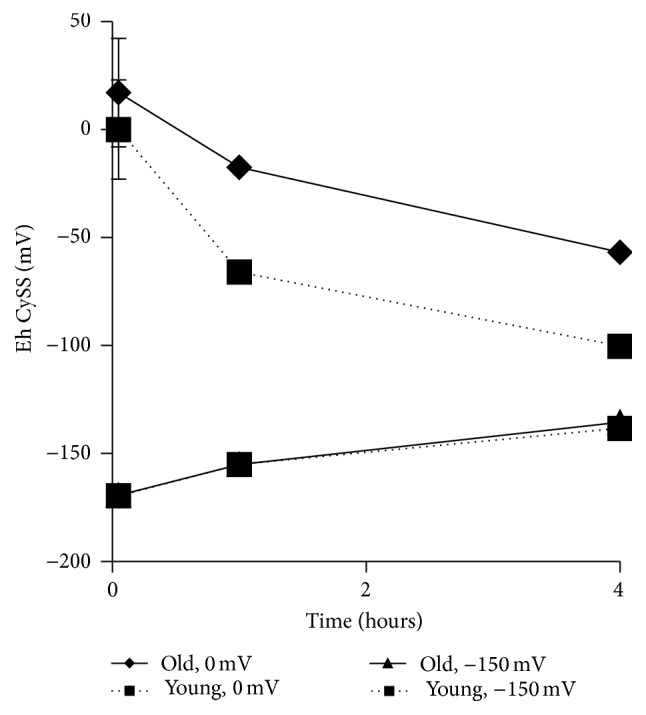
Time course of E_h_CySS restoration by young and old fibroblasts after challenge with oxidizing or reducing media. Lung fibroblasts from young and old mice were incubated with either 0 mV media or −150 mV media for 0, 1, or 4 hours. Conditioned media were removed and analyzed for Cys and CySS concentrations by HPLC.

The data in Figure 1 showed that redox equilibrium is restored within 24 hours after it is perturbed. Here, we examined earlier time points to determine how quickly equilibrium is reestablished and whether there is a difference between old and young cells. When challenged with a reducing environment (−150 mV), both young and old cells moved toward their preferred E_h_CySS set point at equal rates (Figure 4). In contrast, the response of young and old cells to an oxidizing environment was different: by 1 hour, young cells had nearly reached their set point while old cells were only just beginning to reduce the medium. By 4 hours, however, both young and old cells had restored their media to 95 mV and −60 mV, respectively. When compared with Figure 1, it can be seen that these respective E_h_CySS values were maintained for at least 24 hours.

### 3.4. The CySS Transporter Is Downregulated in Old Fibroblasts

**Figure 5:**
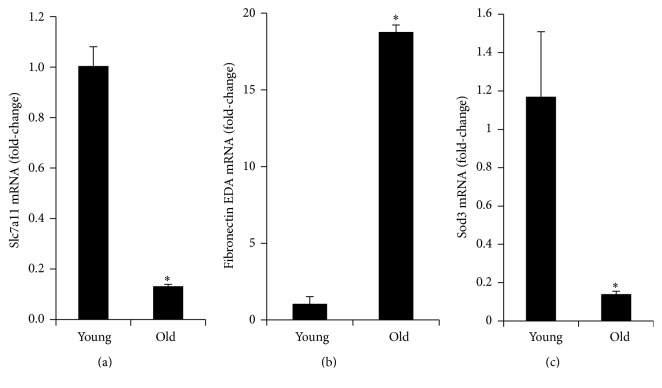
Expression levels of the CySS transporter subunit Slc7a11 and the fibronectin EDA splice variant in young and old fibroblasts. Lung fibroblasts from young and old mice were plated in 6-well plates at a density of 800,000 cells per well in complete DMEM. Cells were collected 24 hours later, and RNA was isolated for RT-PCR analysis. (a) Decreased expression of Slc7a11 in lung fibroblasts harvested from aged animals when compared to those obtained from young animals. (b) Increased expression of fibronectin EDA in lung fibroblasts harvested from aged animals when compared to those obtained from young animals (^*∗*^ *p* < 0.001).

The abovementioned data suggest that old fibroblasts may be deficient in cystine transport. To begin to assess this, we examined the expression of the CySS transporter, specifically the Slc7a11 subunit of the xCT transporter that confers specificity [36]. As predicted from the observed decreased rate of CySS uptake, there was 10-fold lower expression of Slc7a11 in lung fibroblasts harvested from aged animals when compared to those obtained from young animals (Figure 5(a)).

### 3.5. Aging Is Not Associated with Global Changes in Expression of Antioxidant and Thiol-Regulating Enzymes

To further assess changes that occurred with aging, microarray analysis was used to identify genes that were differentially expressed in fibroblasts as a function of age.  Table 1 lists genes that could contribute to differences in the extracellular redox states through their roles as Cys and CySS transporters and glutathione-related thiol-disulfide regulating enzymes.  Table 2 lists thioredoxin-related thiol-disulfide regulating enzymes and antioxidant defense enzymes. Remarkably, only 2 of these genes exhibited greater than 2-fold difference between young and old cells: Slc7a11 and Sod3. As discussed above, Slc7a11 encodes the CySS- and Glu-specific subunit of the xCT transport system. Sod3 encodes the extracellular superoxide dismutase that catalyzes the dismutation of superoxide anion to hydrogen peroxide and molecular oxygen. If the stringency of the comparison is lowered by removing the fold-change cut-off and relying solely on the adjusted *p* value to assign significance, 6 more genes are added to the list. Of these, Slc1a4 and Glx are downregulated in aging fibroblasts, whereas the other 4 are upregulated, and, therefore, would not explain the increase in extracellular redox potential.

### 3.6. Old Fibroblasts Express Higher Levels of Fibronectin EDA and Other Components of the Extracellular Matrix

Like aging, culturing fibroblasts in oxidizing media promote their transdifferentiation into myofibroblasts, characterized by increased expression of fibronectin (Fn1), transforming growth factor-*β* (Tgfb1), and *α*-smooth muscle actin (Acta2) [30, 37]. Because old fibroblasts naturally condition their media to a more oxidizing state than young fibroblasts (see Figure 1(a)), we reasoned that these cells would express higher levels of Fn1 and its profibrotic splice variant FnEDA. Indeed, old fibroblasts had 18-fold higher levels of fibronectin EDA mRNA than young fibroblasts (Figure 5(b)). Examination of the microarray data confirmed that aging fibroblasts had increased expression of Fn1, Tgfb1, and Acta2 (Table 3). In addition, 4 different collagen genes and Tgfb3 were also upregulated (Table 3). Two laminins that have been reported to be downregulated in the aging lung, Lama3 and Lama4 [38], were downregulated in the fibroblasts from old mice. A third laminin, Lama5, was upregulated in fibroblasts from old mice (Table 3). Thus, production of extracellular matrix components can be affected by either artificial manipulation of the extracellular E_h_CySS [30] or age-related differences in the set point of the extracellular E_h_CySS.

## 4. Discussion

In the current study, we found that primary lung fibroblasts from young mice condition their media close to −95 mV within 4 hours of a media change. This value is within the range of −90 to −100 mV reported for mouse plasma E_h_CySS [39–41] but is more reduced than the average value of −80 mV observed in human plasma [24, 26, 42–44]. These data support the concept that mice and humans have different optimal plasma E_h_CySS set points and that cells derived from these species reproduce these set points when placed in culture. When human cancer cell lines HT-29 and Caco-2 are grown in culture, they condition their extracellular E_h_CySS to −80 mV, and stimulation with growth factors increases the rate at which they achieve homeostasis [27, 45, 46]. In a study of murine bone marrow derived dendritic cells, Yan et al. found that the cells reached an extracellular E_h_CySS of −110 mV, but only when stimulated to proliferate by coculturing with T cells [29]. The primary mouse fibroblasts used in the present study were actively proliferating and rapidly achieved an extracellular redox potential that was consistent with those observed in mice and mouse cell cultures, but that was more reducing than those observed in humans and human cell cultures.

Plasma redox potentials become more oxidizing with age, but the reasons for this are unclear. We now report that cells isolated from an old mouse retain an apparent preference for an oxidizing extracellular environment or are unable to create an optimally reducing environment. We found that fibroblasts from old mice expressed much lower levels of the CySS-specific transporter subunit Slc7a11 than fibroblasts from young mice. This finding was consistent with a decreased rate of CySS removal from the media and a decreased rate of reduction of an oxidizing environment. To metabolize extracellular CySS to Cys, the cells must import CySS, reduce it to Cys [47], then export Cys back into the extracellular space. At least 2 enzymes have been shown to have CySS-reducing activity: thioredoxin 1 (Txn1) and thioredoxin domain containing 17 (Txndc17), both of which receive electrons from thioredoxin reductase (Txnrd1) [47]. However, expression of the genes encoding these enzymes was unchanged in aging fibroblasts. Therefore, our data support the interpretation that import of CySS, rather than reduction of CySS to Cys, is impaired in aging cells. However, it will be important to determine whether the activities, not just mRNA levels, of these CySS reductases remain unchanged in aging fibroblasts.

The expression of extracellular Sod3 was also lower in old fibroblasts than in young fibroblasts. Loss of this important antioxidant from the extracellular compartment could contribute to the shift toward more oxidizing conditions that we observed in this study. The slower rate of CySS decline from the conditioned media of old fibroblasts relative to young fibroblasts could be the result of either a slower rate of cellular uptake (by Slc7a11) or an increased rate of its formation via oxidation of Cys. Cu,Zn-SOD (Sod1) can catalyze the oxidation of Cys in the presence of oxygen [48], but the decrease in expression of extracellular Cu,Zn-SOD (Sod3) that we observed does not support this mechanism in aging. It is possible that the amount of SOD protein or SOD activity is increased in the extracellular compartment of aging fibroblasts, despite the observed decrease in expression. Future studies will need to investigate this potential mechanism. Alternatively, increased extracellular superoxide concentrations as a result of lower extracellular Cu,Zn-SOD activity could lead to increased rates of Cys oxidation via direct or indirect reactions [49]. However, we did not observe a corresponding increase in the rate of Cys disappearance from the media, supporting the conclusion that differences in CySS uptake are one of the major forces leading to changes in steady state CySS concentrations between young and old cells.

While our results point to decreased expression of the CySS transporter as one of the major factors leading to oxidation of the extracellular redox state of aging cells, there are other potential mechanisms that could be contributing. For example, mitochondrial production of superoxide, hydrogen peroxide, and other oxidizing species tends to increase with age [50]. Increased oxidant production within the mitochondria can lead to oxidation of cytoplasmic targets [51], but it is unclear what effect this would have on extracellular redox state. An increased oxidant burden in the cytoplasmic compartment could decrease the rate of CySS reduction to Cys, consistent with results of the present study showing that extracellular Cys concentrations are lower in cultures of fibroblasts from old mice. Further studies will be needed to assess the relative contributions of decreased import of extracellular CySS and decreased reduction of intracellular CySS in regulating the balance between CySS and Cys in the extracellular space.

Cells not only regulate their extracellular environment but also respond to it. In the present study, we incubated cells in media with E_h_CySS values ranging from 0 mV to −150 mV to investigate how well cells restored the E_h_CySS to their preferred set point. We and others have used a similar approach to demonstrate that incubating cells in different redox media affects proliferation [46], cell adhesion [52], inflammatory signaling [53–55], cancer cell invasiveness [56, 57], membrane receptor activation [58], and expression of extracellular matrix proteins [30]. For example, adding a 0 mV medium to cells results in higher expression of genes indicative of myofibroblast differentiation than adding −150 mV medium. The present study shows that cells from an old mouse produce an extracellular redox environment that mimics this effect of artificially adding an oxidizing environment. Therefore, cells are not just passively responding to changes in extracellular E_h_CySS; they are changing the extracellular E_h_CySS. Interestingly, the cells become more myofibroblast-like in an oxidized extracellular environment regardless of whether the oxidation arises as a result of external forces or by its own manipulation of the environment.

## 5. Conclusions

Fibroblasts play a key role in remodeling the extracellular matrix in response to stress, but it is unknown how they contribute to extracellular redox remodeling. Here, we show that fibroblasts play an active role in controlling their extracellular redox environment and that fibroblasts from old mice are either unable to attain an ideal E_h_CySS or have been reprogrammed to maintain a more oxidizing set point. These results may explain the subtle profibrotic remodeling of the extracellular matrix that occurs with aging and lead to a better understanding of how changing extracellular redox potential increases susceptibility to lung injury.

## Figures and Tables

**Table 1:** Differential expression of genes related to Cys and CySS transporters and glutathione-related thiol-disulfide enzymes.

Gene	Old versus young (fold-difference)^a^	Step-up *p* value^b^
*Cys and CySS transporters*		
**Slc7a11**	**Down 5.0-fold**	**0.0034**
Slc7a9		0.28
Slc1a1		0.85
Slc1a4	Down 1.5-fold	**0.034**
Slc1a5		0.092
Slc3a1		0.38
Slc3a2		0.69

*Thiol-disulfide enzymes: Glutathione-related*		
Gpx1	Up 1.4-fold	**0.033**
Gpx2		0.096
Gpx3		0.49
Gpx4		0.064
Gpx5		0.87
Gpx6		0.71
Gpx7		0.067
Gpx8		0.23
Gsr		0.54
Gclc		0.62
Gclm		0.60
Gss		0.48
Glrx	Down 1.7-fold	**0.0097**
Glrx2		0.11
Glrx3	Up 1.4-fold	**0.044**
Glrx5	Up 1.2-fold	**0.048**

^a^Fold-difference in expression in old fibroblasts relative to the expression in young fibroblasts is shown for all genes with a significant difference. Those exceeding the cut-off threshold of 2-fold are shown in bold.

^b^Step-up false discovery rate for the comparison between young and old cells following correction for multiple comparisons.

**Table 2:** Differential expression of genes related to thioredoxin-related thiol-disulfide enzymes and antioxidant enzymes.

Gene	Old versus young (fold-difference)^a^	Step-up *p* value^b^
*Thiol-disulfide enzymes: thioredoxin-related*		
Prxd1		0.62
Prdx2		0.35
Prdx3	Up 1.4-fold	**0.0074**
Prdx4		0.20
Prdx5		0.29
Prdx6		0.40
Prdx6b		0.93
Srxn1		0.64
Txn1		0.77
Txn2		0.79
Txnrd1		0.65
Txnrd2		0.91
Txnrd3		0.25
Txndc17		0.24
Txnip		0.09

*Antioxidant enzymes*		
Sod1		0.32
Sod2		0.17
**Sod3**	**Down 4.1-fold**	**0.0074**
Cat		0.75
G6pd2		0.99

^a^Fold-difference in expression in old fibroblasts relative to the expression in young fibroblasts is shown for all genes with a significant difference. Those exceeding the cut-off threshold of 2-fold are shown in bold.

^b^Step-up false discovery rate for the comparison between young and old cells following correction for multiple comparisons.

**Table 3:** Differential expression of genes related to the extracellular matrix in primary lung fibroblasts from young and old mice. Only genes that were significantly different between old and young cells are shown.

Gene	Old versus young (fold-difference)^a^	Step-up *p* value^b^
*Myofibroblast transdifferentiation*		
Tgfb1	Up 1.6-fold	**0.038**
**Tgfb3**	**Up 6.0-fold**	**0.0033**
**Acta2**	**Up 2.2-fold**	**0.022**
Fn1	Up 1.3-fold	**0.0091**
**Col4a1**	**Up 3.2-fold**	**0.016**
**Col4a2**	**Up 2.3-fold**	**0.0096**
Col5a2	Up 1.3-fold	**0.048**
**Col11a1**	**Up 3.5-fold**	**0.0033**
Lama3	Down 1.3-fold	**0.023**
**Lama4**	**Down 16.4-fold**	**0.0016**
**Lama5**	**Up 6.0-fold**	**0.0014**

^a^Fold-difference in expression in old fibroblasts relative to the expression in young fibroblasts is shown for all genes with a significant difference. Those exceeding the cut-off threshold of 2-fold are shown in bold.

^b^Step-up false discovery rate for the comparison between young and old cells following correction for multiple comparisons.
